# Disulfiram Exerts Antifibrotic and Anti-Inflammatory Therapeutic Effects on Perimysial Orbital Fibroblasts in Graves’ Orbitopathy

**DOI:** 10.3390/ijms23095261

**Published:** 2022-05-09

**Authors:** Xing Wang, Huijing Ye, Shenglan Yang, Xiaotong Sha, Xiandai Wang, Te Zhang, Rongxin Chen, Wei Xiao, Huasheng Yang

**Affiliations:** State Key Laboratory of Ophthalmology, Zhongshan Ophthalmic Center, Sun Yat-sen University, Guangdong Provincial Key Laboratory of Ophthalmology and Visual Science, Guangzhou 510060, China; wangx659@mail2.sysu.edu.cn (X.W.); yehuijing@gzzoc.com (H.Y.); yshlan@mail2.sysu.edu.cn (S.Y.); shaxt@mail2.sysu.edu.cn (X.S.); wangxd202204@163.com (X.W.); zhangt257@mail2.sysu.edu.cn (T.Z.); chenrongxin@gzzoc.com (R.C.); xiaowei@gzzoc.com (W.X.)

**Keywords:** disulfiram, Graves’ orbitopathy, perimysial orbital fibroblasts, myofibroblasts, fibrosis

## Abstract

Fibrosis of extraocular muscles (EOMs) is a marker of end-stage in Graves’ orbitopathy (GO). To determine the antifibrotic and anti-inflammatory therapeutic effects and the underlying molecular mechanisms of disulfiram (DSF) on perimysial orbital fibroblasts (pOFs) in a GO model in vitro, primary cultures of pOFs from eight patients with GO and six subjects without GO (NG) were established. CCK-8 and EdU assays, IF, qPCR, WB, three-dimensional collagen gel contraction assays, cell scratch experiments, and ELISAs were performed. After TGF-β1 stimulation of pOFs, the proliferation rate of the GO group but not the NG group increased significantly. DSF dose-dependently inhibited the proliferation, contraction, and migration of pOFs in the GO group. Additionally, DSF dose-dependently inhibited fibrosis and extracellular matrix production markers (FN1, COL1A1, α-SMA, CTGF) at the mRNA and protein levels. Furthermore, DSF mediates antifibrotic effects on GO pOFs partially through the ERK-Snail signaling pathway. In addition, DSF attenuated HA production and suppressed inflammatory chemokine molecule expression induced by TGF-β1 in GO pOFs. In this in vitro study, we demonstrate the inhibitory effect of DSF on pOFs fibrosis in GO, HA production, and inflammation. DSF may be a potential drug candidate for preventing and treating tissue fibrosis in GO.

## 1. Introduction

Graves’ orbitopathy (GO) is an ocular manifestation of Graves’ disease in orbital connective tissue, and extraocular muscle (EOM) fibrosis is a hallmark of end-stage inflammation and tissue remodeling. Imaging shows single or multiple EOMs with spindle-shaped hypertrophy (abnormal thickening of the muscle belly). Loss of normal contractile function increased orbital pressure, interorbital crowding, and compression of the optic nerve lead to diplopia, restrictive strabismus, ocular motility disorders, dysthyroid optic neuropathy (DON), impaired visual function, and even blindness. The quality of life of patients with this condition is seriously affected, and the prognosis is poor. In a randomized controlled study of DON [1], 45% of the patients in the glucocorticoids (GCs)—only intravenous shock treatment group had no improvement in visual acuity compared with 82% of the patients in the surgery-only treatment group, indicating the importance of early intervention for disease progression to DON.

The pathological histology of GO is characterized by inflammatory edema of the orbital soft tissues and EOMs in the early stages. Myofibroblasts (MFs) are observed in mid-to-late-stage EOMs, with widened muscle gaps, structural disruption of muscle fibers, collagen deposition, interstitial fibrosis, and muscle fibrosis contractures. It is too late to treat EOM fibrosis when serious complications arise. Therefore, we need to study the pathophysiological process in-depth to identify new targets for early intervention.

Orbital fibroblasts (OFs) can be functionally divided into two cell subtypes based on the differential expression of the Thy-1 antigen, with anatomical and biosynthetic heterogeneity. Thy-1(+) fibroblasts transform into MFs, while Thy-1(−) fibroblasts transform into mature adipocytes. In orbital adipose/connective tissue from patients with GO, only approximately 50–70% are Thy-1(+) fibroblasts. However, in perimysial orbital fibroblasts (pOFs) from patients with GO or control EOM tissue, all cells uniformly expressed Thy-1 with a 100% positivity rate [2]. Patients with GO had enhanced expression of Thy-1(+) in OFs [3]. Transforming growth factor-β (TGF-β), as a pivotal factor in the initiation and development of fibrosis, plays a vital role in the transition of OFs and the development of GO-related fibrosis [4,5]. When exposed to high concentrations of TGF-β, Thy-1(+) OFs differentiate into MFs. MFs have morphological characteristics of both fibroblasts and smooth muscle cells. These cells show strong secretion, proliferation, migration, and contraction and produce high levels of extracellular matrix (mucopolysaccharide, hyaluronic acid, etc.) and fibrous collagen, ultimately leading to scar formation and tissue fibrosis. To date, most researchers attribute GO-related muscle dysfunction in EOMs to the malignant behavior of pOFs. With pOFs as the target, studying the pathophysiological mechanism of TGF-β in the development of EOM fibrosis will help identify new targets for GO treatment.

Disulfiram (DSF), a member of the dithiocarbamate family, has been approved by the Food and Drug Administration (FDA) to treat alcohol addiction since 1951 [6]. Its good pharmacokinetics, safety, and tolerance in humans have been widely recognized in clinical practice. A growing body of evidence suggests that this old clinical drug has new applications in the future. Studies have shown that DSF can benefit by reducing inflammation and fibrosis in various diseases. DSF significantly reduced liver inflammation, suppressed inflammation-related genes, inhibited macrophage infiltration, attenuated hepatic steatosis, and substantially reduced liver fibrosis by regulating lipid metabolism and oxidative stress [7]. Moreover, DSF was reported to contribute to increased cancer cell death and tumor tissue necrosis via the ROS/MAPK and ferroptosis pathways. DSF combined with copper inhibited TGF-β1-induced α-SMA expression and suppressed fibroblast activation [8]. DSF decreased the expression of GSDMD and downregulated the level of α-SMA in renal tissues, inhibiting pyroptosis and improving renal fibrosis in rats [9]. In a retrospective cohort study, administration of DSF was found to reduce the risk of SARS-CoV-2 infection with a risk ratio of 0.66 (34% risk reduction) and the severity of COVID-19. There were no COVID-19-related deaths among the 188 SARS-CoV-2-positive patients treated with DSF [10]. DSF was shown to reduce neutrophil extracellular trap (NET) formation, improve blood oxygenation, reduce pulmonary edema, reduce perivascular fibrosis, and improve survival rates [11]. DSF has been relatively little studied in ocular inflammation and fibrosis. Previously, DSF was shown to significantly reduce collagen levels and decrease cell proliferation rates by inhibiting ALDH1 in ocular mucous membrane pemphigoid (OMMP) and conjunctival scarring, effectively reducing ocular surface inflammation scores and preventing local fibrosis [12].

Based on these mechanisms, we hypothesized that DSF would have beneficial effects on pOFs in GO and can be further developed as a GO treatment. Here, we first report the antifibrotic effects of DSF on GO in an in vitro model and explore the underlying mechanisms. In addition, we found that DSF could attenuate HA production and downregulate inflammatory and chemokine expression.

## 2. Results

### 2.1. DSF Inhibits TGF-β1-Induced Proliferation of GO pOFs

**Figure 1:**
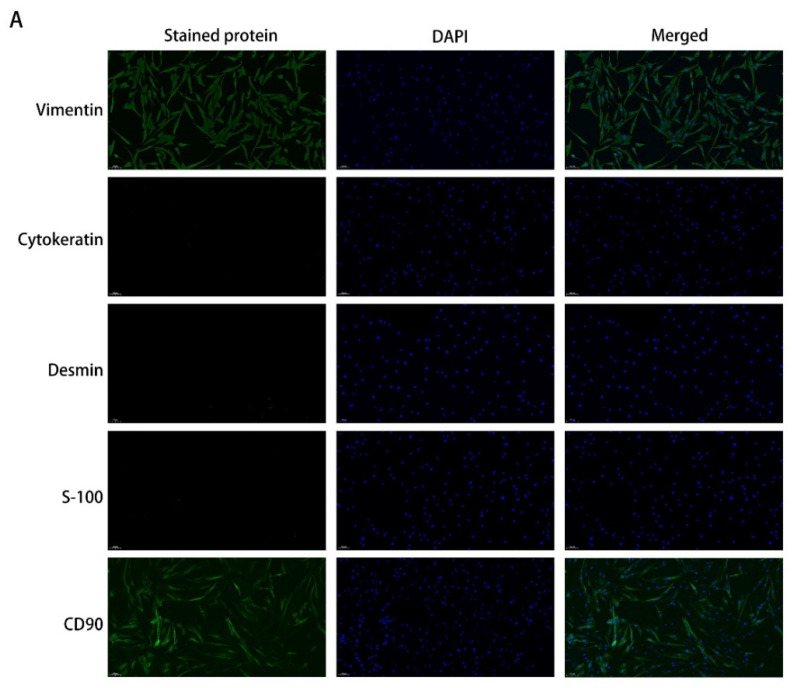

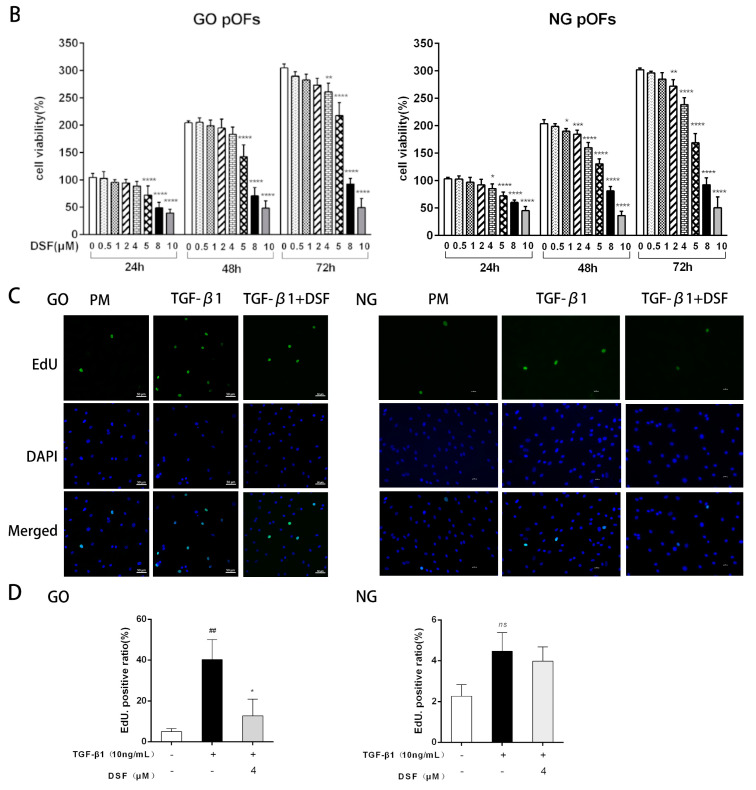
DSF inhibits TGF-β1-induced cell proliferation in the pOF model of GO. (**A**) IF staining of pOFs with vimentin, CD90, cytokeratin, desmin, and S-100 antibodies (green). Cell nuclei were stained with DAPI (blue). The stained cells were examined under a fluorescence microscope (100×); scale bar = 100 μm. (**B**) pOFs in the GO and NG groups were treated with increasing concentrations of DSF (0, 0.5, 1, 2, 4, 5, 8, and 10 μM) in PM for 24 h, 48 h, and 72 h. Cell viability is presented as the percentage relative to the viability of the untreated cells. (**C**) Representative images of the EdU incorporation assay results in the pOFs in the GO and NG groups treated with TGF-β1/DSF at the indicated concentrations. The cells were observed using a fluorescence microscope (200×), scale bar = 50 μm. Green: EdU, blue: DAPI. (**D**) Quantification of the EdU incorporation assay results of the pOFs in the GO and NG groups (control, TGF-β1 (10 ng/mL), TGF-β1 + DSF (4 μM)), *n* = 5. The data are expressed as the triplicates’ mean ± standard deviation (SD). ^##^ *p* < 0.01 compared with the control; * *p* < 0.05, ** *p* < 0.01, *** *p* < 0.001, and **** *p* < 0.0001 compared with TGF-β1 alone; ns denotes no statistical significance versus the control/TGF-β1; assessed by one-way ANOVA.

The third-generation cells of the GO and NG groups of EOMs were long and shuttle-shaped, with round or oval nuclei. Uniformly high expression of the fibroblast marker proteins vimentin and CD90 in the cytoplasm and negative expression of cytokeratin, desmin, and S-100 were found. This finding indicates that the primary cells obtained in this experiment are fibroblasts consistent with an EOM origin (Figure 1A). To determine the noncytotoxic DSF concentrations, we performed CCK-8 assays of pOFs. The results showed that DSF at concentrations <5 μM was safe for pOFs in the GO and NG groups (Figure 1B). To further assess whether DSF affects the proliferation of pOFs in the presence of TGF-β1, we performed EdU assays at 24 h of TGF-β1 stimulation of the pOFs in the GO and NG groups. Interestingly, we found a higher rate of EdU positivity in the GO group (4.977 ± 1.46%) than in the NG group (2.62 ± 0.98%) at the PM baseline level, which is consistent with the previous reports [13]. Cell division was accelerated in the GO group pOFs when TGF-β1 was stimulated alone but not significantly in the NG group pOFs. Therefore, all subsequent experiments were completed in the GO group pOFs. The EdU-positive rate of the cells was significantly lower in the GO group pOFs after DSF pretreatment (Figure 1C,D).

### 2.2. DSF Exerts Antifibrotic Effects on pOFs in the GO Group

**Figure 2:**
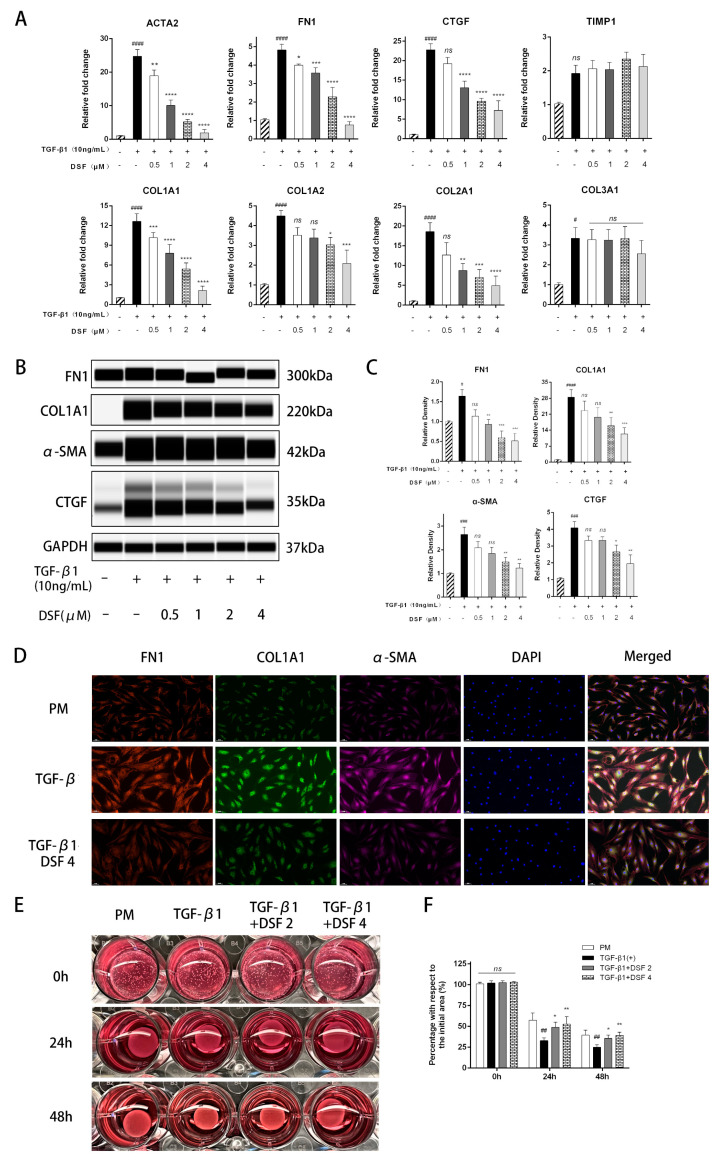

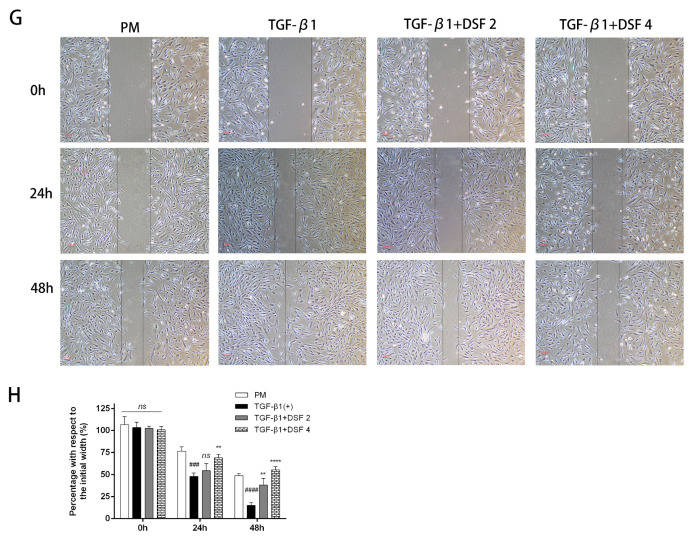
DSF exerts antifibrotic effects on pOFs in the GO group. (**A**) The mRNA levels of fibrotic and extracellular matrix production markers (ACTA2, FN1, CTGF, TIMP-1, COL1A1, COL1A2, COL2A1, and COL3A1) were measured, *n* = 5. (**B**) The protein expression levels of the indicated fibrotic markers in each group were assessed. (**C**) The protein levels were quantified, analyzed, and normalized to the level of GAPDH for each sample, *n* = 3. (**D**) IF staining of pOFs with FN1 (red), COL1A1 (green), and α-SMA (pink) antibodies after treatment with TGF-β1 (10 ng/mL)/DSF (4 μM). Cell nuclei were stained with DAPI (blue). The stained cells were examined under a fluorescence microscope (200×); scale bar = 50 μm. (**E**) Photographed collagen gel contraction of pOFs after treatment with TGF-β1 (10 ng/mL)/DSF (2 μM, 4 μM) for 0 h, 24 h, and 48 h. (**F**) Statistical analysis of the percentage with respect to the initial area, *n* = 3. (**G**) Photographed wound repairability of pOFs after treatment with TGF-β1 (10 ng/mL)/DSF (2 μM, 4 μM). Scale bar = 100 μm. (**H**) Statistical analysis of the rate of wound closure, *n* = 3. The data are expressed as the triplicates’ mean ± standard deviation (SD). ^#^ *p* < 0.05, ^##^ *p* < 0.01, ^###^ *p* < 0.001, and ^####^ *p* < 0.0001 compared with the control; * *p* < 0.05, ** *p* < 0.01, *** *p* < 0.001, and **** *p* < 0.0001 compared with TGF-β1 alone; ns denotes no statistical significance versus the control/TGF-β1; assessed by one-way ANOVA.

MFs constitute a major source of extracellular matrix production, are involved in the evolution from the inflammatory state to the repair and fibrotic state and are essential target cells for controlling fibrosis progression [14]. TGF-β significantly promotes the proliferation and conversion of OFs to MFs and regulates the expression of TSHR [15]. First, we measured the mRNA expression of markers associated with fibrosis and extracellular matrix production. pOFs treated with 10 ng/mL TGF-β1 for 48 h exhibited increased ACTA2, FN1, CTGF, COL1A1, COL1A2, COL2A1, and COL3A1 levels but no significant change in TIMP-1. Furthermore, cotreatment with DSF significantly downregulated the levels of these markers (Figure 2A). At the protein level, pOFs also showed similar phenomena. DSF inhibited the levels of FN1, COL1A1, α-SMA, and CTGF in a dose-dependent manner (Figure 2B,C). As shown in cellular IF staining, the cell morphology of the TGF-β1-stimulated group was larger and more plumped up than that of the control group, with an increase in cell peduncles and high expression of α-SMA, FN1, and COL1A1, indicating that the pOFs had differentiated into MFs. DSF pretreatment showed a significant inhibitory effect (Figure 2D).

Collagen gel contraction is a suitable model in vitro that can simulate the cellular contraction process in tissue in three dimensions. As shown in Figure 2E,F, TGF-β1 promoted the contraction of collagen-containing pOFs. The collagen area gradually decreased with time in all groups. However, the degree of contraction in the group treated with DSF was significantly lower than that in the group stimulated with TGF-β1 alone, indicating an inhibitory effect on collagen contraction. In addition, the impact of DSF on pOF migration in the GO group was assessed by scratch wound assays. We observed delayed wound closure in the DSF-treated groups compared with the untreated groups. Over time, quantitation of wound widths revealed a significant inhibitory effect of DSF on OF motility in the GO group (Figure 2G,H).

### 2.3. DSF Regulates the ERK-Snail Pathway during Fibrosis

**Figure 3:**
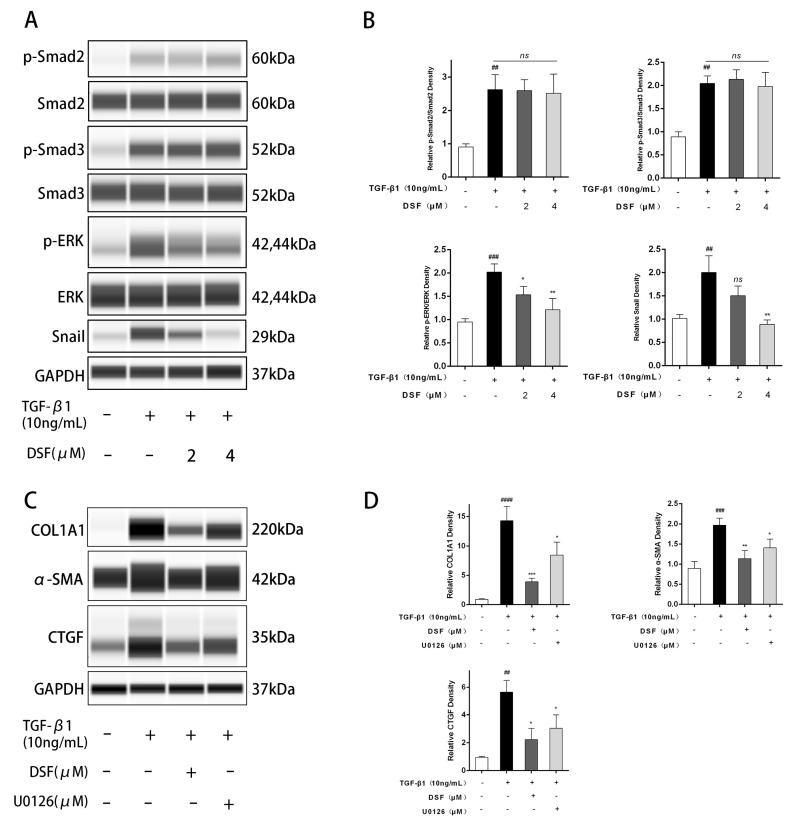

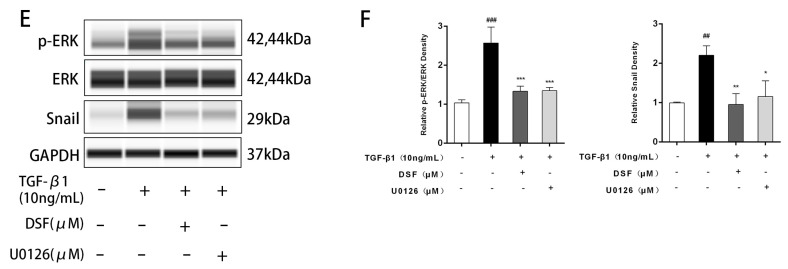
DSF regulates the ERK-Snail pathway during fibrosis. (**A**) Cell lysates were subjected to Western blotting, and p-Smad2, Smad2, p-Smad3, Smad3, p-ERK, ERK, and Snail expression were assessed. (**B**) Protein expression levels of p-Smad2/Smad2, p-Smad3/Smad3, p-ERK/ERK, and Snail were quantified by densitometry and normalized to the GAPDH level for each sample, *n* = 3. (**C**) The protein expression levels after treatment with TGF-β1 (10 ng/mL)/DSF (4 μM)/U0126 (10 μM). (**E**) p-ERK, ERK, and Snail expression were assessed by Western blotting. (**D**,**F**) The protein levels were quantified, analyzed, and normalized to the level of GAPDH for each sample, *n* = 3. The data are expressed as the triplicates’ mean ± standard deviation (SD). ^##^ *p* < 0.01, ^###^ *p* < 0.001, and ^####^ *p* <0.0001 compared with the control; * *p* < 0.05, ** *p* < 0.01, and *** *p* < 0.001, compared with TGF-β1 alone; ns denotes no statistical significance versus TGF-β1 alone.

To further elucidate the mechanism through which DSF inhibits proteins related to fibrosis and the extracellular matrix, we detected proteins in the related pathways by WES. The results showed that the expression of the classic proteins Smad2 and Smad3 was significantly upregulated after TGF-β1 stimulation, and DSF pretreatment did not affect the expression of Smad2 and Smad3 (Figure 3A,B). The MAPK cascade response has previously been shown to play an essential role in apoptosis and cytokine expression, with the classic ERK cascade playing a central role in mitosis, cell differentiation, and cell proliferation [16]. ERK phosphorylation levels were increased after TGF-β1 stimulation, and DSF dose-dependently inhibited p-ERK/ERK levels (Figure 3A,B). Moreover, as an essential transcription factor in the process of cell proliferation and migration, Snail expression was reported to be upregulated by ERK and TGF-β [17]. We found that Snail levels were upregulated after TGF-β1 stimulation and that DSF dose-dependently inhibited Snail protein expression (Figure 3A,B).

Then, we selected the ERK1/2 inhibitor U0126 (Cell Signaling Technology, Boston, MA, USA, #9903S) for further validation using a concentration (10 μM) obtained in previous literature. The results showed that U0126 also inhibited the expression of α-SMA, CTGF, and COL1A1, but its effect was not as strong as that of DSF (Figure 3C,D). U0126 significantly inhibited the phosphorylation of ERK and downregulated Snail expression (Figure 3E,F). This finding suggests that there may be other pathways by which DSF inhibits MF transformation and extracellular matrix production.

### 2.4. DSF Attenuates HA Production and Suppresses Inflammatory and Chemokine Expression

**Figure 4:**
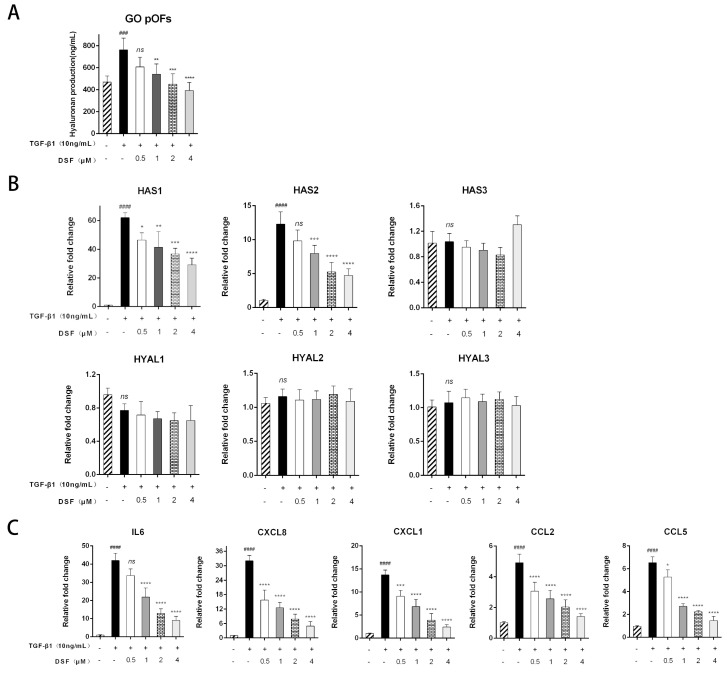
DSF attenuates HA production and suppresses inflammatory molecule expression. (**A**) The concentrations of HA in the GO pOF cell supernatants were quantified by ELISAs. (**B**) mRNA expression levels of HAS1, HAS2, HAS3, HYAL1, HYAL2, and HYAL3. (**C**) mRNA levels of IL-6, CXCL8, CXCL1, CCL2, and CCL5. Each graph shows the mean ± SD of repeated experiments from at least 3 patients. ^###^ *p* < 0.001, ^####^ *p* < 0.0001 compared with the control; * *p* < 0.05, ** *p* < 0.01, *** *p* < 0.001, and **** *p* < 0.0001 compared with TGF-β1 alone; ns denotes no statistical significance versus the control/TGF-β1.

HA is the primary glycosaminoglycan that plays an essential role in the extracellular matrix [18]. We investigated whether DSF inhibited HA production in primary cultures of pOFs via ELISAs. As shown in Figure 4A, TGF-β1 increased HA production in the GO group pOFs compared to the untreated control cells. Additionally, pretreatment with DSF decreased HA production in a dose-dependent manner. Changes in the levels of mRNAs and core genes associated with HA (HAS1, HAS2) confirmed these results (Figure 4B). However, the mRNA expression levels of HAS3, HYAL1, HYAL2, and HYAL3 were not affected by TGF-β1 or DSF. Furthermore, we found that DSF significantly suppressed the IL-1β-induced mRNA expression of inflammatory molecules, including IL-6, CXCL8, CXCL1, CCL2, and CCL5 (Figure 4C).

## 3. Discussion

The clinical treatment of GO involves a comprehensive treatment model combining drugs and surgery based on the severity and activity of the disease. There is no effective non-surgical treatment for inactive fibrotic GO. Surgery can only reduce strabismus and relieve optic nerve compression but cannot treat the lesioned muscle itself. Surgery should not be performed until the disease is stable and the inflammation has been extinguished. Moreover, the risk of multiple surgeries, surgical complications, and mental and economic stress are all issues. Therefore, it is important to study the mechanisms related to EOM fibrosis and to explore effective prevention and treatment methods.

Novel biologics, such as teprotumumab [19,20], rituximab [21], adalimumab [22], infliximab [23], tocilizumab [24,25], secukinumab [26], and imatinib [27], have been reported in the GO field. These drugs only have partial therapeutic effects in improving GO inflammation, proptosis, etc. However, there are few prospective, multicenter, clinical randomized controlled studies on drug efficacy, safety, optimal dosage, and optimal use. In addition, the drugs (pirfenidone [28], quercetin [29], simvastatin [30], curcumin [31], and PH20 [32]) for the treatment of GO fibrosis were tested in vitro. In the current study, we demonstrate the inhibitory effect of DSF on GO pOF fibrosis. Furthermore, DSF potently suppressed HA production and exerted anti-inflammatory effects in vitro. To the best of our knowledge, this is the first study to investigate the therapeutic effect of DSF on GO pOFs.

Almost all fibroblasts derived from the perimysium of EOMs were positive for Thy-1 on the surface. Therefore, the selection of fibroblasts derived from the EOM in the study of the pathogenesis of GO fibrosis is more specific. We found that in TGF-β1-stimulated pOFs, cell proliferation and contractility were enhanced, and the mRNA and protein expression of fibrosis and extracellular matrix markers were significantly enhanced. Our experiments demonstrated for the first time that DSF in pOFs can dose-dependently inhibit TGF-β1-induced cell proliferation and migration, reduce the protein expression of FN1, COL1A1, α SMA, and CTGF, inhibit the differentiation of pOFs to MFs, and suppress extracellular matrix production.

We found that cell replication and division rates simultaneously increased in the TGF-β1-stimulated GO pOFs, but not in the NG pOFs. Increased production of hydrogen peroxide can stimulate the proliferation of GO OFs and induce the production of proinflammatory cytokines [33]. In addition, the mRNA and protein levels of the platelet-derived growth factor AB and BB dimers were increased in orbital tissue in the GO group compared with the NG group; these dimers play essential roles in stimulating OF proliferation, activation, and HA production [34]. Immunohistochemistry of EOMs has also shown that α-SMA and collagen are overexpressed in GO samples compared with NG samples [32]. This evidence suggests that GO pOFs may be more easily transformed into MFs than NG pOFs and have stronger proliferative capacity under the same TGF-β1 stimulation conditions.

ERK activation was shown to be essential in TGF-β-induced epithelial–mesenchymal transition (EMT) and is required for the breakdown of adherens junctions and cell motility [35]. In NMuMG cell lines, TGF-β1 stimulation significantly induced ERK phosphorylation, and treatment with the ERK1/2 inhibitor U0126 inhibited this phosphorylation and kinase activity, blocking TGF-β1-induced EMT [36]. As a transcription factor, Snail plays a crucial role in embryonic development and cancer progression by mediating EMT [37]. Cyclovirobuxine D (an alkaloid derived from *Populus tremula*) could inhibit EMT, proliferation, and invasion of tumor cells through the CTHRC1-AKT/ERK-Snail signaling pathway and exert anticancer effects [38]. These studies suggest that activation of the ERK signaling pathway plays a vital role in cell proliferation and migration and can act upstream of Snail. Our study also found that the expression of α-SMA, CTGF, and COL1A1 could be downregulated after the addition of the ERK1/2 inhibitor U0126. Moreover, U0126 downregulated Snail expression levels, confirming that DSF inhibits TGF-β1-induced transformation of MFs and extracellular matrix production partially through the ERK-Snail signaling pathway. The aberrant regulation of lysyl oxidase (LOX) family enzymes is involved in the overproduction of extracellular matrix. Since DSF can also exert antifibrotic effects by inhibiting LOX/LOXL2 [39] and pyroptosis [9] pathways, whether these processes are also present in pOFs still needs to be investigated in depth.

A study showed that the expression of IL-1β, IL-6, IL-18, and TNF-α in peripheral blood and renal tissues of rats with unilateral ureteral obstruction was significantly reduced by DSF [9]. In a mouse model of lung injury associated with severe acute pancreatitis, DSF was found to inhibit the cleavage of GSDMD, reduce the expression levels of proinflammatory cytokines (IL-1β and IL-18), and significantly decrease the levels of lipase, amylase, TNF-α, and IL-6, ameliorating pancreatic tissue injury and reducing lung inflammation [40]. Our study also found that DSF inhibited TGF-β1-induced secretion of inflammatory markers and chemokines (IL6, CXCL8, CXCL1, CCL2, and CCL5) and reduced HA production. The specific mechanism of action of this anti-inflammatory effect of DSF in the GO in vitro model will be investigated in our next study.

### Study Limitations

There are several limitations in the present study. First, our experiments were only in vitro and did not verify whether DSF can also improve the inflammation and fibrosis of EOMs in an animal model of GO. Second, DSF is a clinical oral drug. Due to the lack of a stable animal model of GO, the systemic effects (thyroid function indicators, liver and kidney function, etc.) and adverse reactions of DSF for GO treatment cannot be determined. Third, the number of clinical specimens used in the experiment was small. EOM tissue samples could be collected from GO patients undergoing strabismus surgery. The sample size needs to be expanded to verify the experimental results. Finally, due to the limited amount of EOM tissue obtained during surgery, we did not conduct in-depth research on the EOM tissue itself, such as gene sequencing and immunohistochemistry. We plan to collect more samples in our follow-up experiments, further elucidate the pathogenesis, and identify new therapeutic targets.

## 4. Materials and Methods

### 4.1. Primary Cultures of pOFs

EOM tissue samples were collected from eight patients with GO who underwent orbital decompression surgery. All cases were euthyroid during the operation. Non-GO control (NG) samples were collected from six patients without GO who underwent enucleation. The clinical patient information is provided in Table 1. Patients who received GCs systemic/local within 3 months were excluded. The severity and the clinical activity of GO were graded according to the NOSPECS classification (0 = no symptoms or signs; I = only signs, no symptoms; II = soft tissue involvement; III = proptosis; IV = EOM involvement; V = corneal involvement; VI = sight loss, due to optic nerve involvement) and the seven-item clinical activity score (CAS) scheme proposed by EUGOGO, respectively [41,42,43]. All patients signed informed consent forms. This study was conducted according to the Declaration of Helsinki and approved by the Institutional Review Board of Zhongshan Ophthalmic Center (2016KYPJ028, 20 July 2017).

After removal of fascia and extensive vascular tissue, tissue explants were cut into small pieces and plated in 10 cm culture dishes in high-glucose Dulbecco’s Modified Eagle’s Medium (DMEM, #C11965500BT) containing 20% fetal bovine serum (FBS, #10270-106-1) and 1% penicillin/streptomycin (#15140122) (all from Gibco Laboratories, New York, NY, USA). After the cells had migrated out of the tissue pieces and reached confluence, they were passaged with 0.25% trypsin/EDTA (Gibco, New York, NY, USA, #25200072). Then, the cells were grown in proliferation medium (PM) (DMEM containing 10% FBS and 1% penicillin/streptomycin) using standard cell culture protocols. For the experiments, pOFs were at passages 3–7, and each experiment was repeated with at least 3 independent specimens.

### 4.2. Immunofluorescence (IF) Analysis

IF staining of cells was performed as described previously [44]. The primary antibodies included vimentin (#ab92547), CD90 (#ab133350), cytokeratin (#ab76126), desmin (#ab32362), S-100 (#ab109252) (all from Abcam Plc, Cambridge, UK), α-SMA (#19245S), FN1 (#26836S), and COL1A1 (#39952S) (all from Cell Signaling Technology, Boston, MA, USA). The secondary antibody was anti-rabbit/mouse Alexa Fluor Plus 488/594 (Thermo Fisher Scientific, Rockford, IL, USA, #A32731TR, #A32723, #A32740, #A32742). All antibodies were diluted in proportion with NCM Universal Antibody Diluent (NCM Biotech, Suzhou, China, #WB500D). Images were observed by an inverted fluorescence microscope (Nikon, Tokyo, Japan, 100×/200×) and photographed.

### 4.3. Cell Viability Assays

Cell viability was assessed using a Cell Counting Kit-8 (CCK-8, Bimake, Shanghai, China, #B34304) assay. Briefly, pOFs were treated with different concentrations of DSF (Sigma-Aldrich, Shanghai, China, #86720) for the indicated times in 96-well plates. The measurements were similar to those of a previous study [13].

### 4.4. EdU

The pOFs were seeded in a 6-well plate with preinstalled cell slides. Then, medium containing TGF-β1 (10 ng/mL, R&D Systems, Minneapolis, MN, USA, # 240-B-010) and DSF (0, 2 μM, 4 μM) was added, and the cells were cultured for 24 h. DNA synthesis was determined by a 5-ethynyl-2′-deoxyuridine (EdU) incorporation assay using a Cell Light EdU DNA Imaging Kit (Beyotime, Shanghai, China, #C0071S) according to the manufacturer’s protocol and observed by inverted fluorescence microscopy (Nikon, Tokyo, Japan, 200×). The number of positive nuclei/total nuclei was determined using ImageJ software (National Institutes of Health, Bethesda, MA, USA).

### 4.5. Collagen Gel Contraction Assay

The pOFs in the logarithmic growth phase were digested with trypsin to obtain a cell suspension (serum-free medium) of 2.5 × 10^5^ cells/mL, which was placed on ice for later use. Moreover, a type-I collagen solution (Solarbio, Beijing, China, #C8062) was prepared (100 μL of type I collagen, 6 μL of 0.1 M NaOH, and 12 μL of 10× DMEM) and mixed with the 382 μL of cell suspension per well, and then the corresponding stimuli were added. The mixture was incubated on a 24-well plate. Subsequently, the gel was released from the edges with a pipette tip and allowed to contract for a period of 24 h, 48 h, and 72 h. For evaluation of the percentage reduction of the gel area, photographs were taken and analyzed using ImageJ software (National Institutes of Health, Bethesda, MA, USA).

### 4.6. Wound-Healing Assay

Confluent cell monolayers in a 6-well plate were wounded by mechanical scraping; a straight scratch was made with a pipette tip, simulating a wound. Wound width was assessed at the time of scraping to ensure that all wounds were the same width at the beginning of the experiment. The cell culture medium was then replaced with fresh medium with DSF (0, 2 μM, 4 μM) and 10 ng/mL TGF-β1 and incubated for 24 h, 48 h, and 72 h. Phase-contrast microscopy was used to observe wound closure (Nikon, Tokyo, Japan, 40×) and take photographs. The results are expressed as the wound width.

### 4.7. RNA Extraction and Quantitative Real-Time Polymerase Chain Reaction (qPCR)

According to the manufacturer’s protocol, total RNA was extracted using a TaKaRa Mini BEST Universal RNA Extraction Kit (TaKaRa, Dalian, China, #9767). cDNA was synthesized from total RNA using Prime Script RT Master Mix (TaKaRa, Dalian, China, #RR036B). qPCR was carried out on a Roche Light Cycler 480 (Roche, Basel, Switzerland) using TB Green Premix Ex Taq II (TaKaRa, Dalian, China, #RR820B). The primer pair sequences for qPCR are listed in Table 2. GAPDH was used as the housekeeping gene.

### 4.8. Automated Western Blotting

A protein extraction kit (KeyGEN, Nanjing, China, #KGP250, #KGP950) was used to lyse the cells. Protein concentrations were measured with a BCA kit (Beyotime, Shanghai, China, #P0010). Protein separation and detection were performed using an automated capillary electrophoresis system (Simple Western system and Compass software; ProteinSimple, San Jose, CA, USA, Version: 5.0.0). Wes Separation Capillary Cartridges for 12–230/66–440 kDa (ProteinSimple, San Jose, CA, USA, #SM-W004/SM-W008) were used for the proteins. The following primary antibodies were used: GAPDH (#5174S), α-SMA (#19245S), FN1 (#26836S), COL1A1 (#39952S), CTGF (#86641S), Smad2 (#5339S), phospho-Smad2 (p-Smad2, #3108S), Smad3 (#9523S), phospho-Smad3 (p-Smad3, #9520S), ERK (#4695S), phospho-ERK (p-ERK, #4370S), and Snail (#3879S) (all from Cell Signaling Technology, Boston, MA, USA). Signals were detected with an HRP-conjugated anti-rabbit secondary antibody (ProteinSimple, San Jose, CA, USA, #DM-001) and were visualized using Compass for SW software.

### 4.9. Hyaluronan (HA) Measurement

Cells were treated with different concentrations of DSF (0, 0.5, 1, 2, or 4 μM) and 10 ng/mL TGF-β1 in serum-free DMEM. After 24 h, the cell culture media were collected and centrifuged at 5000× *g* for 10 min. According to the manufacturer’s instructions, the concentrations of HA in the cell culture supernatant were quantified with an enzyme-linked immunosorbent assay (ELISA) kit (R&D Systems, Minneapolis, MN, USA, #DHYAL0).

### 4.10. Statistical Analysis

All experiments were performed at least three times with samples from different individuals, and samples were assayed in duplicate each time. The data are expressed as the mean value and standard deviation. All calculations and statistical analyses were performed using GraphPad Prism v9 (GraphPad Software, Inc., La Jolla, CA, USA). Statistical analyses were performed with one-way ANOVA. A *p* value < 0.05 was considered significant.

## 5. Conclusions

GO is a complex disease and seriously affects patient quality of life. The early clinical application of more effective, safer, longer-lasting, and simplified drugs or treatments is needed to improve the long-term prognosis and quality of life of patients with GO to the greatest extent. Our studies in the pOF in vitro model of GO provide strong evidence that DSF inhibits pOFs differentiation into MFs, extracellular matrix generation, and inflammation. In conclusion, we propose that DSF may be a potential drug candidate for preventing and treating tissue fibrosis in GO.

## Figures and Tables

**Table 1:** Clinical features of patient samples used in this study.

Age (Years)	Gender (M/F)	Smoking (Y/N)	Duration of GO (Years)	Previous GO Treatment	Proptosis (R/L, mm)	CAS	GO Severity Assessment	Surgery Performed
GO patients								
64	M	Y	2.25	GCs	20/18	3/7	VI	Decompression
58	F	N	2.5	GCs	19/20	0/7	VI	Decompression
68	M	N	3	None	20/18	1/7	VI	Decompression
47	M	Y	1.75	GCs	18/19	2/7	VI	Decompression
43	M	N	1.25	GCs	20/21	0/7	VI	Decompression
50	F	N	1.5	None	22/22	2/7	IV	Decompression
42	F	N	1	None	19/20	0/7	IV	Decompression
54	M	N	2	GCs	19/19	2/7	VI	Decompression
Non-GO control patients								
45	F	N	-	-	-	-	-	Enucleation
55	M	Y	-	-	-	-	-	Enucleation
51	F	N	-	-	-	-	-	Enucleation
39	F	N	-	-	-	-	-	Enucleation
56	F	N	-	-	-	-	-	Enucleation
67	M	N	-	-	-	-	-	Enucleation

M, male, F, female; Y, yes; N, no; GO, Graves’ orbitopathy; R/L, right or left eyes; CAS, clinical activity score; GCs, glucocorticoids.

**Table 2:** qPCR primer sequences.

Genes	Sequences (5′-3′)
ACTA2	F: GAACCCTAAGGCCAACCGGGAGAAA
	R: CCACATACATGGCGGGGACATTGA
FN1	F: ACAAGCATGTCTCTCTGCCAA
	R: GCAATGTGCAGCCCTCATTT
CTGF	F: AGCTGACCTGGAAGAGAACATT
	R: GCTCGGTATGTCTTCATGCTG
TIMP1	F: CATCACTACCTGCAGTTTTGTG
	R: TGGATAAACAGGGAAACACTGT
COL1A1	F: AAAGATGGACTCAACGGTCTC
	R: CATCGTGAGCCTTCTCTTGAG
COL1A2	F: CTCCATGGTGAGTTTGGTCTC
	R: CTTCCAATAGGACCAGTAGGAC
COL2A1	F: CCAGATGACCTTCCTACGCC
	R: TTCAGGGCAGTGTACGTGAAC
COL3A1	F: CTTCTCTCCAGCCGAGCTTC
	R: CCAGTGTGTTTCGTGCAACC
HAS1	F: GCGGGCTTGTCAGAGCTAC
	R: ACTGCTGCAAGAGGTTATTCC
HAS2	F: CCTCCTGGGTGGTGTGATTT
	R: GCGTCAAAAGCATGACCCAA
HAS3	F: TTATACAGCTTTTCTACCGGGG
	R: CAGAAGGCTGGACATATAGAGG
HYAL1	F: TTCCCTGACTGCTACAACTATG
	R: CATGTAGATGCTGGGATAGAGG
HYAL2	F: CATGATTATGTGCAGAACTGGG
	R: GTCGTGTGAAGACGTAGACTG
HYAL3	F: CAGCTCTACAAGGCCTATACTG
	R:TAGTTGGAAGCCATACTATGCC
IL6	F: CACTGGTCTTTTGGAGTTTGAG
	R: GGACTTTTGTACTCATCTGCAC
CXCL8	F: CCACCGGAAGGAACCATCTC
	R: GGGGTGGAAAGGTTTGGAGT
CXCL1	F: TTCACAGTGTGTGGTCAACAT
	R: AAGCCCCTTTGTTCTAAGCCA
CCL2	F: CCTTCATTCCCCAAGGGCTC
	R: CTTCTTTGGGACACTTGCTGC
CCL5	F: GCAAGCTTTGTCACCCGAAA
	R: CCCAAGCTAGGACAAGAGCA
GAPDH	F: TTGCCATCAATGACCCCTT
	R: CGCCCCACTTGATTTTGGA

F, forward; R, reverse.

## Data Availability

The datasets generated during and/or analyzed during the current study are not publicly available but are available from the corresponding author on reasonable request.

## Abbreviations

GO, Graves’ orbitopathy; EOM, extraocular muscle; DSF, disulfiram; pOFs, perimysial orbital fibroblasts; OFs, orbital fibroblasts; NG, non-GO control patients; DON, dysthyroid optic neuropathy; GCs, glucocorticoids; MFs, myofibroblasts; TGF-β, transforming growth factor-β; PM, proliferation medium; EMT, epithelial–mesenchymal transition; LOX, lysyl oxidase; α-SMA, alpha smooth muscle actin; CTGF, connective tissue growth factor; FN1, fibronectin 1; IL, interleukin; CCL, C-C motif chemokine ligand; CXCL, C-X-C motif chemokine ligand; HA, hyaluronic acid; HAS, hyaluronic synthase; HYAL, hyaluronidase.
